# The macrocyclic peptide rhesus theta defensin 1 activates interferon and antiviral pathways in human monocytes

**DOI:** 10.1093/jleuko/qiaf150

**Published:** 2025-10-25

**Authors:** Prasad Tongaonkar, Katie K Trinh, Jill E Henley, Philip J Sell, Vyoma Thakker, André J Ouellette, Michael E Selsted

**Affiliations:** Department of Pathology and Laboratory Medicine, Keck School of Medicine, University of Southern California, Los Angeles, CA 90033, United States; Department of Pathology and Laboratory Medicine, Keck School of Medicine, University of Southern California, Los Angeles, CA 90033, United States; Biosafety Level 3 Facility in the Hastings Foundation and the Wright Foundation Laboratories, Keck School of Medicine, University of Southern California, Los Angeles, CA 90033, United States; Molecular Microbiology and Immunology, Keck School of Medicine, University of Southern California, Los Angeles, CA 90033, United States; Department of Pathology and Laboratory Medicine, Keck School of Medicine, University of Southern California, Los Angeles, CA 90033, United States; Department of Pathology and Laboratory Medicine, Keck School of Medicine, University of Southern California, Los Angeles, CA 90033, United States; USC Norris Comprehensive Cancer Center, Keck School of Medicine, University of Southern California, Los Angeles, CA 90033, United States; Department of Pathology and Laboratory Medicine, Keck School of Medicine, University of Southern California, Los Angeles, CA 90033, United States; USC Norris Comprehensive Cancer Center, Keck School of Medicine, University of Southern California, Los Angeles, CA 90033, United States

**Keywords:** antiviral, defensin, interferon, monocyte, SARS-CoV-2

## Abstract

Rhesus theta defensin (RTD)-1, a cyclic antimicrobial peptide, regulates gene expression and immune signaling pathways in cell culture and animal models of immune-mediated diseases. In lipopolysaccharide-stimulated cells, RTD-1 inhibition of proinflammatory cytokine secretion and gene expression is mediated through inhibition of the NF-κB and MAP kinase signaling pathways. To gain insights into RTD-1 regulation of naïve cells, we performed RNA sequencing (RNA-seq) to determine the effect of the peptide on global gene expression in human monocytes and THP-1 monocytes. In both cell types, analysis of differentially expressed genes revealed stimulation of interferon and antiviral gene expression pathways. RTD-1 induced Y701 phosphorylation of STAT1 and activated the ISRE reporter in a JAK-dependent manner. Stimulation of the ISRE reporter by RTD-1 was interferon-α/β receptor dependent but was independent of its NF-κB inhibitory activity in lipopolysaccharide-stimulated cells. RTD-1 inhibited infection of vesicular stomatitis virus pseudotyped with G glycoprotein or SARS-CoV-2 spike protein in THP-1 and Vero E6 cells, respectively. RTD-1 also inhibited infection of Calu-3 2B4 cells by SARS-CoV-2 virus, demonstrating antiviral activity of RTD-1 in diverse cell types. These results demonstrate that RTD-1 stimulates interferon and antiviral pathways, potentially priming cells for resistance to viral infection.

## Teaser

RTD-1 stimulates phosphorylation of Y701 residue of STAT1 and activates antiviral and interferon-stimulated gene expression pathways in a type I interferon receptor–dependent manner in human monocytes.

## 1. Introduction

Mammalian defensins are tri-disulfide stabilized host defense peptides that play a major role in innate immunity. Of the 3 structural classes of mammalian defensins (alpha [α]-, beta [β]-, and theta [θ]-defensins), θ-defensins are unique, as they are the only known backbone cyclic peptides in the animal kingdom. They have been identified only in Old World monkeys such as rhesus macaques and baboons.^1–3^ θ-Defensins were first discovered in rhesus macaque leukocyte extracts as broad-spectrum antimicrobial peptides with in vitro antimicrobial activities against bacteria and fungi^1,2,4,5^ and several viruses.^6,7^

In addition to their antimicrobial activities, studies have demonstrated that θ-defensins regulate host immune responses in vitro and in murine disease models. We previously reported that rhesus theta defensin-1 (RTD-1) suppresses secretion of several proinflammatory cytokines by lipopolysaccharide (LPS)-stimulated human leukocytes and reduced lethality in a mouse model of polymicrobial sepsis by suppressing dysregulated proinflammatory cytokines.^8^ In a mouse model of systemic candidemia, RTD-1–mediated fungal clearance was associated with a transient induction of neutrophilia followed by homeostatic modulation of proinflammatory cytokines including TNFα, IL-1β, and IL-17.^9^ These studies demonstrated that RTD-1 induces fungal clearance in a host directed manner, rather than by killing the fungal pathogen, and concomitantly suppressed pathologic inflammation.^9^ Similarly, RTD-1 was effective in a mouse model of SARS-CoV-1 infection wherein the peptide had no direct antiviral activity, but rather suppressed pulmonary inflammation which was accompanied by reduced mortality.^10^

In studies using LPS-stimulated human THP-1 cells, RTD-1 inhibited proinflammatory cytokine gene expression (TNF, IL1B, CXCL8, CCL3, and CCL4) by inhibiting the activation of NF-κB and MAP kinase signaling.^8,11^ RTD-1 also stimulated activation of the PI3K—Akt1 pathway, a negative regulator of the NF-κB and MAP kinase pathways.^11^ Recently, RTD-1 was shown to inhibit expression of the microRNA miR-146a suppressing endotoxin tolerance in THP-1 monocytes.^12^ RTD-1 suppresses TNF secretion by reversible inhibition of ADAM17/TACE, which is the primary sheddase that releases TNFα from the cell surface. The cyclic structure of RTD-1 is required for this activity, and the inhibition of ADAM17/TACE is abrogated in the noncyclic isoform of RTD-1.^13^ Consistent with this anti-TNF effect, RTD-1 was highly effective in rat pristane–induced arthritis,^14^ a widely used model of rheumatoid arthritis^14–16^ that is a TNF-driven autoimmune disease.

The aforementioned studies demonstrated that RTD-1 regulates a number of inflammatory pathways associated with inflammation and innate immunity. RTD-1 therefore has potential therapeutic applications in human disease and has been evaluated in phase 1 clinical trials (NCT04286789) for safety. To further explore the effects of RTD-1 on immune responses, we performed transcriptomic studies in human cells to identify pathways affected by peptide treatment in naïve mononuclear phagocytes, cells that play a central role in numerous immune responses. Here, we report that RTD-1 activates interferon (IFN) and antiviral pathways and stimulates phosphorylation of STAT1. Consistent with these effects, RTD-1 induces antiviral responses in human monocytes and inhibits SARS-CoV-2 infection of human airway epithelial cells.

## 2. Methods

### 2.1 Cells and reagents

Positively selected CD14+ human monocytes were purchased from Lonza and STEMCELL Technologies. THP-1 cells (ATCC) were grown in RPMI + 10% fetal bovine serum (FBS) + 1% penicillin/streptomycin (P/S) medium at 37 °C and 5% CO_2_. THP-1 Dual cells (InvivoGen) were maintained in culture as recommended by manufacturer. HEK293T cells and Vero E6 cells were obtained from ATCC and grown in Dulbecco's Minimal Essential Medium (DMEM) + 10% FBS + 1% P/S and in Eagle's Minimal Essential Medium (EMEM) + 10% FBS + 1% P/S, respectively, at 37 °C and 5% CO_2_ as recommended by the supplier. Vero E6 cells overexpressing ACE2 (VeroE6-hACE2) were obtained from Dr. Jae Jung and maintained in DMEM high glucose, supplemented with 10% FBS and 2.5 μg/mL puromycin at 37 °C and 5% CO_2_ in a humidified atmosphere. SARS-CoV-2 (USA-WA1/2020) stocks were obtained from BEI Resources (https://www.beiresources.org/) and cultured/passaged 4 times in VeroE6-hACE2 cells and harvested at 48 h postinoculation. No human subjects were involved in the study, and Institutional Review Board approval was not required.

Synthetic RTD-1 (>95% purity) was prepared as described previously^1^ and used as a 1 mg/mL stock dissolved in 0.01% acetic acid (HOAc). *Salmonella enterica* LPS (Sigma-Aldrich) was dissolved in phosphate buffered saline (PBS). Ruxolitinib (Ruxo) was purchased from Cell Signaling Technology and dissolved in dimethyl sulfoxide (DMSO). IFN-β was purchased from R&D Systems and human IgG1 and human anti IFNAR1 antibodies were purchased from Selleck USA.

### 2.2 RNA-seq and data analysis

Human monocytes from 3 donors were individually analyzed. Cells were thawed in RPMI medium containing 10% heat-inactivated (HI) FBS and 1% P/S + 100 μg/mL normocin and harvested by centrifugation at 200 *g* for 8 min. The cells were then resuspended in RPMI medium containing 1% HI FBS and 1% P/S + 100 μg/mL normocin to a concentration of 1 × 10^6^ cells/mL, and 2 mL of resuspended cells were added to wells of a 6-well plate and equilibrated for 2 h at 37 °C and 5% CO_2_. Cells were treated with 10 µg/mL RTD-1 or with 0.01% HOAc (vehicle control) for 4 h at 37 °C and 5% CO_2_ and harvested by centrifugation at 200 *g* for 8 min. For THP-1, cells were resuspended at 3.3 × 10^5^ cells/mL in RPMI + 1% FBS and 1% P/S for 4 h at 37 °C in 5% CO_2_ and then treated with 1, 3, or 10 μg/mL RTD-1 or 0.01% HOAc (vehicle) control for 20 h. Cells were then harvested at 200 *g* for 8 min. RNA was isolated using Quick RNA Miniprep kit (Zymo Research) from 3 experiments. Viability of RTD-1–treated cells under these experimental conditions was comparable to control cells as determined by Cell Titer Glo assay.

RNA from human monocytes was sequenced at the UCLA Technology Center for Genomics and Bioinformatics. Libraries were prepared using Kapa Stranded RNA-seq kit and single read RNA sequencing (1 × 75 bp) was performed on NextSeq500. RNA from THP-1 cells was sequenced by Genewiz LLC. RNA sequencing libraries were prepared from polyA messenger RNA selected on Oligo dT beads. The samples were sequenced on Illumina HiSeq instrument using a 2 × 150 bp paired-end configuration.

Analysis of RNA-seq data was performed using Partek Flow hosted on High Performance Computing Cluster at the USC Center for Advanced Research Computing. For human monocytes, raw sequencing reads were trimmed from 3′ end and aligned to hg38 using STAR 2.7.8.a aligner. The reads were then quantified to Gencode Genes release 42 annotation model using Partek E/M. Normalization was performed using Poscounts, and DESeq2 was used for calculation of differential gene expression. For THP-1 RNA-seq samples, reads were trimmed and aligned as previous. The reads were quantified to Gencode Genes release 40 and normalization was performed using trimmed mean of M values and adding 0.0001. Differentially expressed genes (DEGs) were identified using analysis of variance after setting the experiment as the random factor. Genes with fewer than 5 reads from all samples were excluded and genes were considered differentially regulated if false discovery rate (FDR) <0.05 and fold change was ≤−1.5 or ≥1.5. Partek Flow's General linear model was used for batch removal. The bubble map was plotted using mean for the group summary method, and hierarchical clustering was performed using Euclidean distance and average linkage. Partek Flow Pearson correlation analysis was performed using normalized counts and FDR <0.05 to identify genes that are regulated by RTD-1.

Gene set enrichment analysis (GSEA) was performed using Partek Flow. Ingenuity Pathway Analysis (IPA) (Qiagen) was used for performing Diseases and Functions and Upstream Regulator analyses. Gene expression data for genes with FDR <0.05 was uploaded to IPA and the Diseases and Functions and Upstream Regulator analysis was performed for genes with fold change ≤ −1.5 or ≥1.5 using core analysis of IPA.

### 2.3 Quantitative real-time polymerase chain reaction

First strand cDNA was generated from RNA using the RT^2^ First Strand DNA kit (Qiagen) as per the manufacturer. The cDNA was used for quantitative real-time polymerase chain reaction (qRT-PCR) using RT^2^ SYBR Green Master Mix using validated primer assays from Qiagen (Table S1 for a list of primers) on a Bio-Rad Opus 96 real-time PCR instrument. ACTB was used for normalizing gene expression and fold change was calculated using the 2^−ΔΔCt^ method^17^ either using Excel (Microsoft) or Geneglobe (Qiagen).

### 2.4 Stimulation of STAT1 Y701 phosphorylation by RTD-1

THP-1 cells were resuspended at ∼3.5 × 10^5^ cells/mL in RPMI + 1% FBS + 1% P/S, and 5 mL of cells were incubated in 60 mM dishes for 3 h at 37 °C and 5% CO_2_ incubator, and either DMSO or Ruxo (0.25 μM) were added for 1 h. The cells were then treated with RTD-1 or with vehicle (0.01% HOAc) and incubation continued for 30 min. Cells were harvested by centrifugation at 200 *g* for 8 min and washed with PBS and stored at −80 °C. The cells were thawed, resuspended in cell lysis buffer (Cell Signaling Technology) containing 0.5 mM PMSF, and homogenized using an insulin syringe. The extracts were clarified by centrifugation at 15,000 rpm for 5 min at 4 °C and protein estimation was performed using BCA kit using BSA standards (Thermo Fisher Scientific). Extracts containing equal amount of protein were resolved on SurePAGE BisTris 10% acrylamide gels (GenScript Biotech) and transferred to a nitrocellulose membrane. The membrane was blocked using 5% milk/TTBS buffer (100 mM Tris HCl pH 7.5, 150 mM NaCl, 0.1% Tween 20) and probed overnight at 4 °C with rabbit anti-phospho-STAT1 Y701 antibodies (Cell Signaling Technology; cat# 9167S), washed with TTBS, and probed with goat anti-rabbit IgG HRP conjugate (Cell Signaling Technology; cat# 7074S). The blots were washed with TTBS and developed using chemiluminescence using LumiGLO reagent (Cell Signaling Technology; cat# 7003). The membrane was stripped with 0.2 M glycine/1 M NaCl pH 2.8, washed with TTBS, and probed with mouse anti-ACTB antibodies (Cell Signaling Technology; cat# 3700S), overnight at 4 °C. The membrane was washed with TTBS and probed with horse anti-mouse IgG HRP conjugate (Cell Signaling Technology; cat# 7076S), washed with TTBS, and then processed using chemiluminescence as above. The western blot films were quantified using ImageJ software version 1.51k (National Institutes of Health).

### 2.5 Analysis of ISRE and NF-κB reporters in THP-1 dual cells

THP-1 Dual cells have 2 reporters, Lucia reporter secreted in the medium by activation of ISRE, which is assayed by measuring luminescence using Quanti-Luc assay, and the secreted embryonic alkaline phosphatase secreted in the medium by activation of the NF-κB pathway, which is assayed using Quanti-Blue. THP-1 Dual cells were resuspended in RPMI containing 1% HI FBS and 0.5% P/S + 100 µg/mL normocin medium at 5.0 ×10^5^ cells/mL for 2 h and treated as described in the figure legends and the incubation continued for 18 h at 37 °C, 5% CO_2_. For treatment with Ruxo, either DMSO or the inhibitor was added 1 h prior to addition of RTD-1 and the incubation continued for 18 h. The medium was collected by centrifugation at 5000 *g* for 5 min. Quanti-Luc Plus or Quanti-Luc4 reagent (InvivoGen) was used for Quanti-Luc assays. The medium was diluted 4-fold with Quanti-Luc reagent in a white opaque 96 well plate (Perkin Elmer) and luminescence was measured with 100 mS integration using SpectraMax i3X plate reader (Molecular Devices). For measuring NF-κB activation, 20 μL of medium was added to 180 μL of Quanti-Blue, incubated at 37 °C for 15 to 20 min, and absorbance measured at 625 nm using SpectraMax i3X plate reader.

### 2.6 Vesicular stomatitis virus G glycoprotein and SARS-CoV-2 spike protein pseudotyped virus infection of cells

A working stock of replication incompetent vesicular stomatitis virus (VSV) in which the VSV glycoprotein (VSV-G) gene is replaced with luciferase (Luc) reporter gene (rVSV-ΔG-Luc) pseudotyped with VSV-G (G*-rVSV-ΔG-Luc) or with SARS-CoV2-S (CoV-2-S*-rVSV-ΔG-Luc) were generated as described previously.^18^ Briefly, HEK293T cells were transfected with pCAGGS-VSV-G or with pCAGGS-SARS-CoV-2 spike (S) plasmids and the cells were then infected with G*-rVSV-ΔG-Luc and incubated for 24 h (virus and plasmid were gifts from Dr. Jae Jung). The media containing G*-rVSV-ΔG-Luc or S*-rVSV-ΔG-Luc pseudotyped viruses were then collected. The median tissue culture infectious dose (TCID50) for G*-rVSV-ΔG-Luc was estimated using HEK293T cells and using Vero E6 cells for CoV-2-S*-rVSV-ΔG-Luc.

THP-1 cells were suspended at 5.0 × 10^5^ cells/mL in phenol red–free RPMI containing 1% FBS + P/S, and 90 µL of the suspension was added to wells of 96 well opaque white plate. After incubation for 2 h at 37 °C, 10 μL of medium containing 10 × vehicle (0.01% HOAc) or indicated concentrations of RTD-1 was added to the wells and incubated for 4 h at 37 °C. Cells were then infected with G*-rVSV-ΔG-Luc (5 µL; TCID50 ∼ 3.16 × 10^4^/10 µL) and incubated at 37 °C and 5% CO_2_ overnight. A similar protocol was used to preincubate G*-rVSV-ΔG-Luc for 90 min with 1, 3, and 10 µg/mL (final concentrations) of RTD-1 at room temperature, after which the mixture was added to wells containing THP-1 cells and incubated overnight at 37 °C. Viral titers were determined by measuring luminescence following addition of 100 µL BriteLite Plus reagent (Perkin Elmer) on a Molecular Devices SpectraMax i3X plate reader with a 100 mS integration time. Assays were performed in triplicate.

Cultured Vero E6 cells were detached using trypsin/EDTA, harvested by centrifugation at 200 *g* for 6 min and resuspended in fresh EMEM + 10% FBS + P/S medium at a concentration of 5 × 10^4^ cells/mL, and 1 × 10^4^ cells (200 µL) were transferred to wells of an opaque white 96-well plate and grown overnight at 37 °C, 5% CO_2_. The medium was then removed and serum-free EMEM medium, RTD-1, and SARS-CoV-2 S protein pseudotyped virus (CoV-2-S*-rVSV-ΔG-Luc; TCID50/mL ∼1.58 × 10^4^), 10 µL diluted 3-fold with 10% FBS/DMEM medium (30 µL), were added to the cells to a total volume of 200 µL. The assay was performed in triplicate. As vehicle controls, 0.01% HOAc and 10% FBS in DMEM were used in place of RTD-1 and CoV-2-S*-rVSV-ΔG-Luc, respectively. To test for potential enzymatic inhibition of luciferase by RTD-1, 2 sets of identical infections were set up and 2 μL of 1 mg/mL RTD-1 (final 10 µg/mL) was added to one set and incubation was continued for another 12 min. The medium was removed and 180 µL of BriteLite Plus diluted with equal volume of D-PBS containing 1 mM MgCl_2_ and CaCl_2_ was added to the wells, and the luminescence was then measured using a SpectraMax i3x plate reader.

### 2.7 SARS-CoV-2 stock and titer determination

SARS-CoV-2 viral cultures were titered by plaque assay. Briefly, a monolayer of VeroE6-hACE2 cells were infected with a serial dilution of viral stocks for 1 h. The viral supernatant was removed, and an overlay of semi-solid agarose/media was added. At day 3 postinfection, plaques were fixed with 4% paraformaldehyde. Postfixation, the agarose/media layer was removed. Plaque visualization was done by staining with 0.2% (w/v) crystal violet solution and plaques were counted to determine plaque forming units. Viral stocks were stored at −80 °C. All experiments with SARS-CoV-2 were performed in Biosafety Level 3 Core Facility in the Hastings Foundation and the Wright Foundation Laboratories facility at the University of Southern California using appropriate personal protective equipment.

### 2.8 Infection of Calu-3 2B4 cells with SARS-CoV-2

Human airway epithelial (Calu-3 2B4) cells, a gift from Dr. Paul McCray, were grown in EMEM medium containing 10% FBS and 1% P/S. Cells were cultured overnight at 5 × 10^5^ cells/mL in a 12-well plate at 37 °C and 5% CO_2_. Medium was then removed and replaced with 1 mL EMEM medium containing 1% FBS and 1% P/S and incubated for 2 h at 37 °C, 5% CO_2_. Medium was then replaced with 1 mL medium containing 0.01% HOAc (control) or RTD-1 and 5 × 10^4^ plaque-forming units (multiplicity of infection 0.1) of SARS-CoV-2 was added and cells incubated for 24 h at 37 °C and 5% CO_2_. The medium was then removed, and cells were solubilized in 0.5 mL TRIzol. Isolation of RNA was performed using Direct-zol RNA miniprep kits (Zymo Research) per the manufacturer's instructions. First-strand cDNA was prepared using the RT^2^ first-strand cDNA kit as described previously. SARS-CoV-2 infection was evaluated by qRT-PCR using primers V1_forward (ATGCTGCAATCGTGCTACAA) and V1_reverse (CCTCTGCTCCCTTCTGCGTA) for the nucleocapsid phosphoprotein (N) gene. RT^2^ SYBR Green qPCR Master Mix was used for qRT-PCR and SARS-CoV-2 N gene expression was normalized to ACTB gene expression and fold change calculated using the −ΔΔC_T_ method.

### 2.9 Statistical analysis

Two-tailed *t* tests were performed using Excel as described in the figure legends. Differences were considered statistically significant if *P* < 0.05.

## 3. Results

### 3.1 RTD-1 stimulates IFN and antiviral pathways in human monocytes

**Fig. 1.**
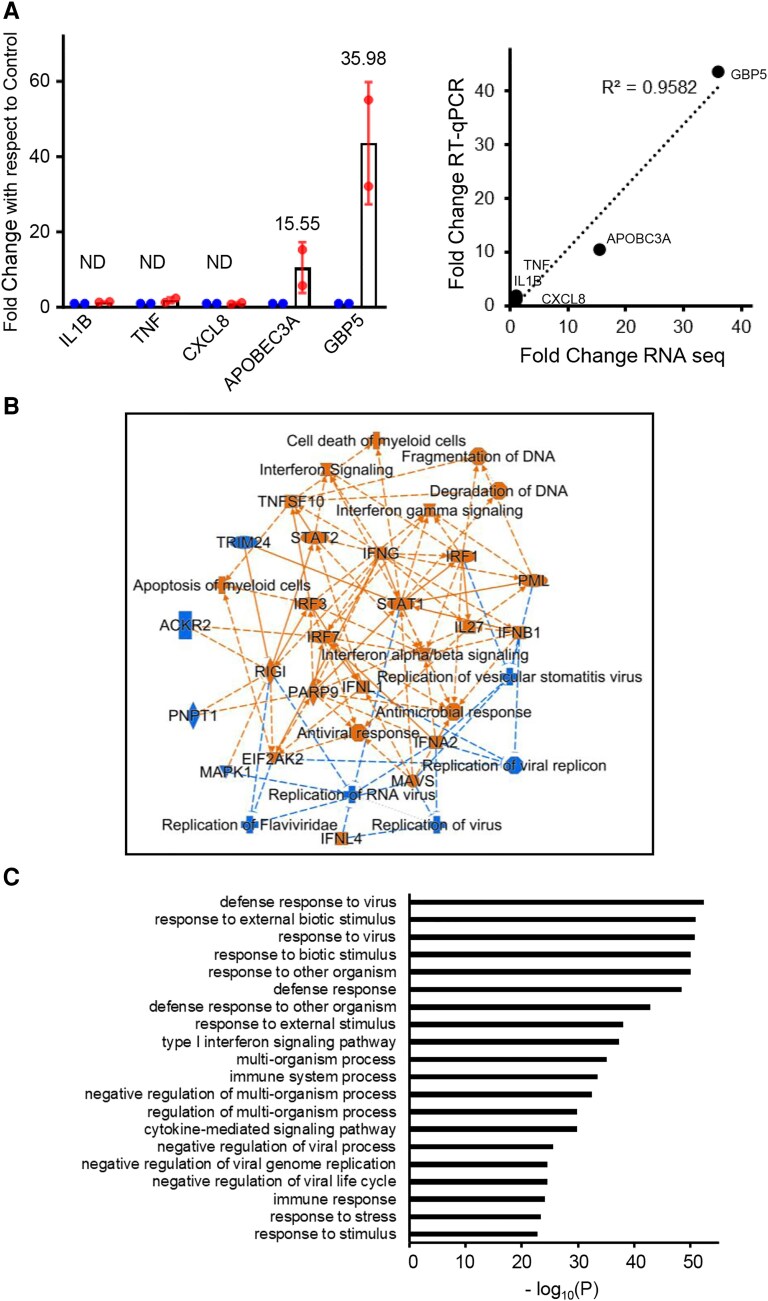
Effect of RTD-1 on gene expression in human monocytes. (A) Validation of RNA-seq analysis. qRT-PCR analysis was performed for select genes from monocytes treated with or without RTD-1. (Left) Gene expression was normalized to ACTB expression, and fold change was calculated with respect to control cells (data from 2 control and 2 RTD-1–treated monocyte samples). Blue: control (0.01% HOAC), red: 10 µg/mL RTD-1 treatment. The numbers indicate change in gene expression observed by RNA-seq analysis. (Right) Comparison of fold change observed by RNA-seq and qRT-PCR for genes in the left panel. (B) IPA generated graphical representation of the RTD-1–induced gene expression in human monocytes. The position of the nodes was tweaked for readability. Blue indicates inhibition and orange indicates activation, and the legend for IPA shapes is available at https://qiagen.my.salesforce-sites.com/KnowledgeBase/articles/Knowledge/Legend. (C) GSEA of changes in gene expression induced by RTD-1. ND, not differentially expressed in RNA-seq.

To investigate the effect of RTD-1 on naïve mononuclear leukocytes, human monocytes were treated with 10 µg/mL RTD-1, a peptide concentration previously shown to modulate inflammatory gene expression in LPS-stimulated cells.^8–11^ RNA-seq analysis disclosed 251 DEGs in peptide-treated cells compared with controls (Table S2). Analysis of selected genes (DEGs and non-DEGs) by qRT-PCR was used to validate RNA-seq results. APOBEC3A and GBP5, upregulated ca. 15- and 35-fold respectively in RNA-seq analyses, were found to be upregulated 10- and 43-fold, respectively, over control gene expression by qRT-PCR analysis (Fig. 1A). RNA-seq of IL1B, TNF, and CXCL8 genes, which are downregulated by RTD-1 in LPS-stimulated THP-1 cells,^11^ was not affected by RTD-1 in naïve monocytes, in agreement with qRT-PCR results (Fig. 1A).

IPA of the 251 DEGs revealed activation of IFN and antiviral pathways including IFN signaling, IFN-γ signaling, and antiviral response, whereas inhibition was observed in pathways such as replication of viral replicon, replication of virus, replication of flaviviridae, replication of RNA virus, and replication of VSV (Fig. 1B). GSEA analysis of the RTD-1–regulated DEGs revealed enrichment of genes involved in defense response to virus, response to virus, type I IFN signaling pathway, and negative regulation of viral process among the top 20 enriched gene sets (Fig. 1C). These results revealed that RTD-1 stimulates IFN and antiviral gene pathways in human monocytes.

**Fig. 2.**
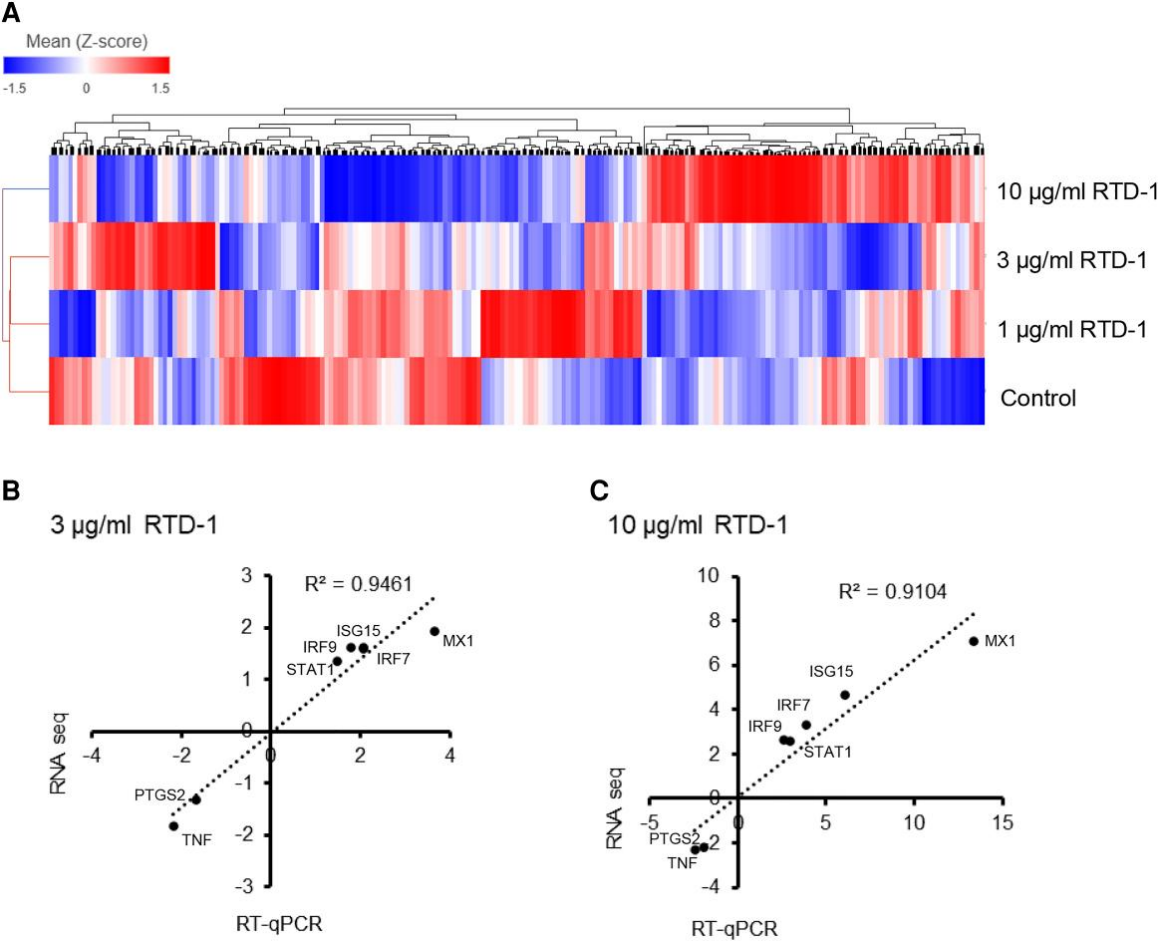
Regulation of gene expression by RTD-1 in THP-1 cells. (A) THP-1 cells were treated with RTD-1 or with 0.01% HOAc vehicle control as indicated and the RNA-seq data from 3 experiments was analyzed by hierarchical clustering. Validation of RNA-seq results using qRT-PCR. Gene expression observed in RNA-seq analysis was evaluated for a few genes using qRT-PCR. The gene expression was normalized to ACTB gene expression and fold change was calculated with respect to the vehicle control. Correlation between qRT-PCR and RNA-seq data (B) with 3 µg/mL RTD-1 and (C)with 10 µg/mL RTD-1.

Because our previous studies demonstrated immunomodulatory effects of RTD-1 on immune-stimulated THP-1 cells, we used this cell line to conduct additional studies on the effect of RTD-1 on gene expression in naïve cells. THP-1 monocytes were treated with 0, 1, 3, or 10 μg/mL RTD-1 for 20 h and analyzed by RNA-seq. As shown in Fig. 2A, hierarchical clustering analysis showed RTD-1 concentration–dependent alterations in gene expression. Interestingly, no DEGs were identified with 1 μg/mL RTD-1 treatment, whereas 9 and 143 DEGs were identified with 3 and 10 μg/mL RTD-1 treatment, respectively (Table S3). The RNA-seq results were validated using qRT-PCR for selected DEGs, which revealed excellent correlation (R^2^ > 0.9) between the RNA-seq and qRT-PCR results (Fig. 2B, C). In addition, gene expression correlation analyses, evaluating the effects of 0, 1, 3, or 10 µg/mL RTD-1 treatments, identified 652 positively correlated genes and 273 negatively correlated genes (FDR < 0.05) (Table S4). Among the RTD-1–correlated genes, positively correlated genes included SHISA5, ATP10A, XAF1, and HELZ2, whereas expression of genes EIF3L, ZSCAN30, and EPAS1 correlated negatively with RTD-1 treatment concentration. Of these SHISA5,^19^ XAF1,^20^ and HELZ2^21^ are involved in antiviral response. The translation initiation factor gene, EIF3L, is known to promote viral translation^22^ and was negatively correlated.

**Fig. 3.**
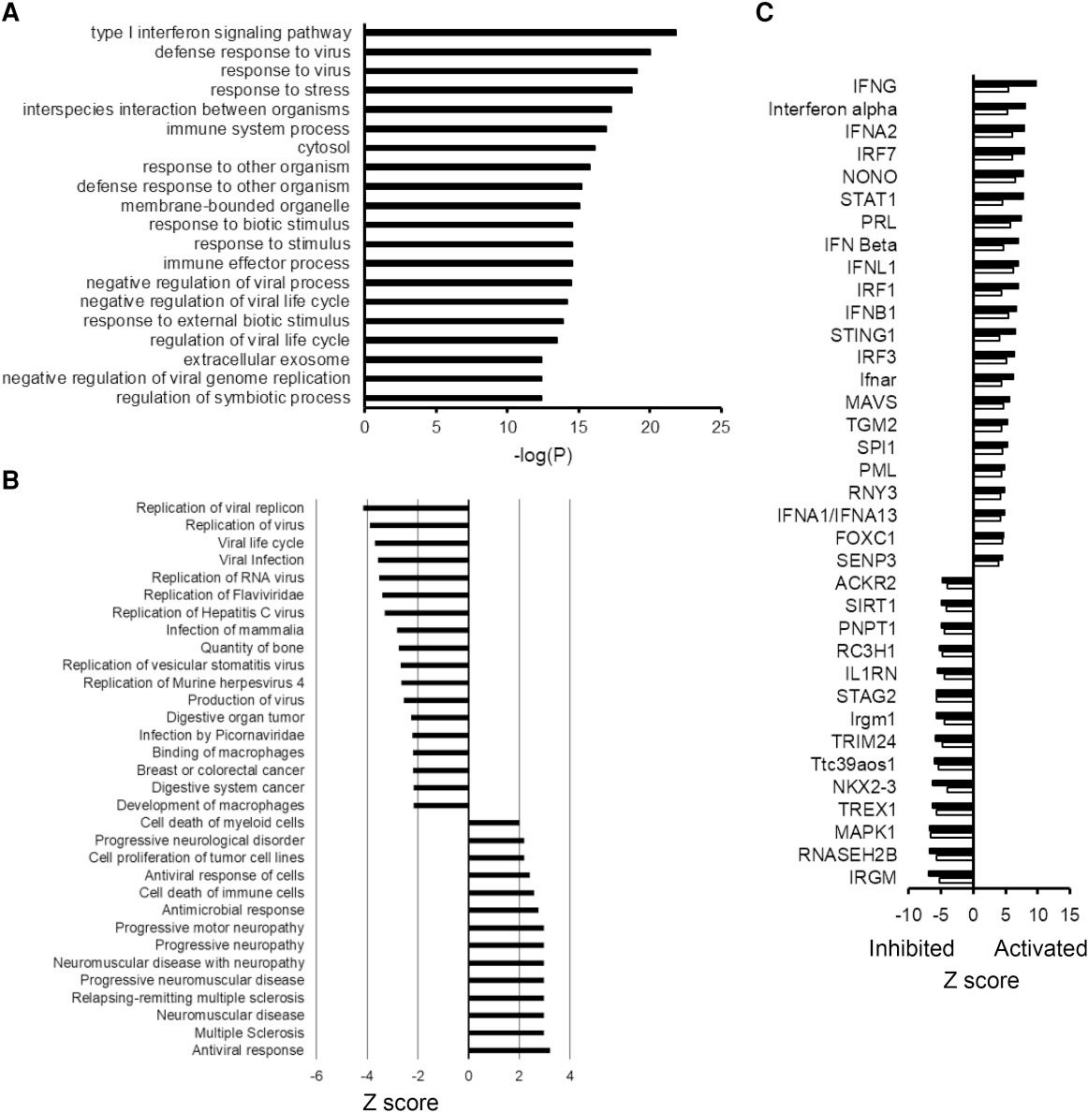
RTD-1 stimulates antiviral and IFN signaling pathways in THP-1 cells. (A) GSEA of 925 genes whose expression is correlated with RTD-1 treatment, and the top 20 enriched groups are shown. (B) IPA Diseases and Functions analysis of DEGs observed in 10 μg/mL RTD-1–treated THP-1 cells. (C) Comparison of IPA upstream regulators observed after 10 µg/mL RTD-1 treatment in monocytes (black bars) and THP-1 cells (white bars) with *z* score ≤−4 or ≥4 for both cell types.

GSEA of RTD-1 correlated genes disclosed an enrichment of type I IFN signaling pathway, viral response pathways, and immune system process (Fig. 3A). Consistent with the GSEA results, Diseases and Functions Analysis of the 143 DEGs observed with 10 μg/mL RTD-1–treated cells disclosed activation of antiviral and antimicrobial responses and inhibition of viral life cycle, viral infection and viral replication pathways (Fig. 3B; Table S5). Upstream regulator analysis of the 143 DEGs (10 μg/mL RTD-1–treated cells compared with control cells) obtained with IPA revealed activation of genes predominantly involved in IFN response (Table S6). Of the activated upstream regulators, IRF7, STAT1, EIF2AK2, DDX58, PARP9, and IRF9 were differentially expressed with more than 2-fold upregulation compared with untreated cells. Based on the expression of the target genes, the upstream regulators SP110, ISG15, ETV7, and USP18 were predicted to be inhibited despite being up regulated >2-fold at the gene expression level, suggesting that there are other pathways involved in regulation of the downstream target genes (Table S6). While the IFN response pathways were activated, the expression of proinflammatory TNF and PTGS2 genes was downregulated by RTD-1 in THP-1 cells consistent with RTD-1 suppressing the basal level expression of these genes.

IPA core analyses of human monocytes and THP1 cells treated with 10 μg/mL RTD-1 showed similar activation states of Upstream Regulators and Diseases and Functions categories in both cells. For example, upstream regulators such as IFNG, IFN-α, IRF7, STAT1 STING1, MAVS were highly activated in both cell types (Fig. 3C;  Table S7). Similarly, RTD-1 activated Diseases and Functions categories including antiviral response, antimicrobial response and other categories including replication of viral replicon and viral life cycle were inhibited in both cell types (Table S8).

### 3.2 RTD-1 stimulates STAT1 phosphorylation

**Fig. 4.**
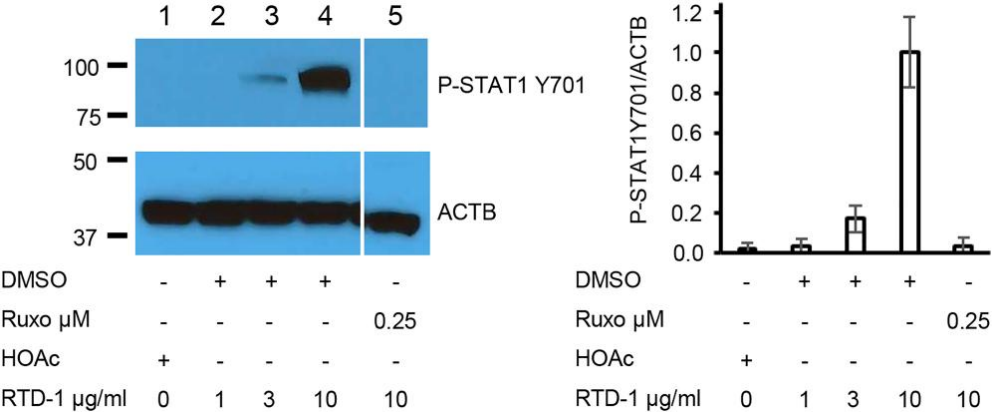
RTD-1 induced phosphorylation of STAT1. THP-1 cells were pretreated with DMSO or Ruxo for 1 h and then with 0.01% HOAc or RTD-1 as shown. The cells were harvested after 30 min and extracts were analyzed by Western blotting experiments using anti-phospho-STAT1 Y701 antibody or anti-ACTB antibody. (Left) One representative gel from 2 experiments. (Right) Ratio of quantification of the phospho-STAT1 Y701 and ACTB bands from Western blots was plotted with respect to RTD-1 concentration. Results are average of 2 experiments. Error bars indicate standard deviation.

Phosphorylation of STAT1 at the Y701 residue plays a major role in IFN-dependent^23,24^ and IFN-independent^25^ pathways for activation of ISRE genes. Therefore, we evaluated the effect of RTD-1 on STAT1 phosphorylation. RTD-1 induced a concentration-dependent increase in Y701 phosphorylation of STAT1 (Fig. 4, left) with ∼40-fold increase in phosphorylation after treatment for 30 min with 10 μg/mL RTD-1 (Fig. 4, compare lanes 3 and 4 with lane 1). This RTD-1–mediated phosphorylation is JAK dependent because pretreatment of cells with Ruxo, a JAK inhibitor, abolished this phosphorylation of STAT1 (Fig. 4, compare lane 5 with lane 4).

### 3.3 RTD-1 stimulates ISRE promoter activity

**Fig. 5.**
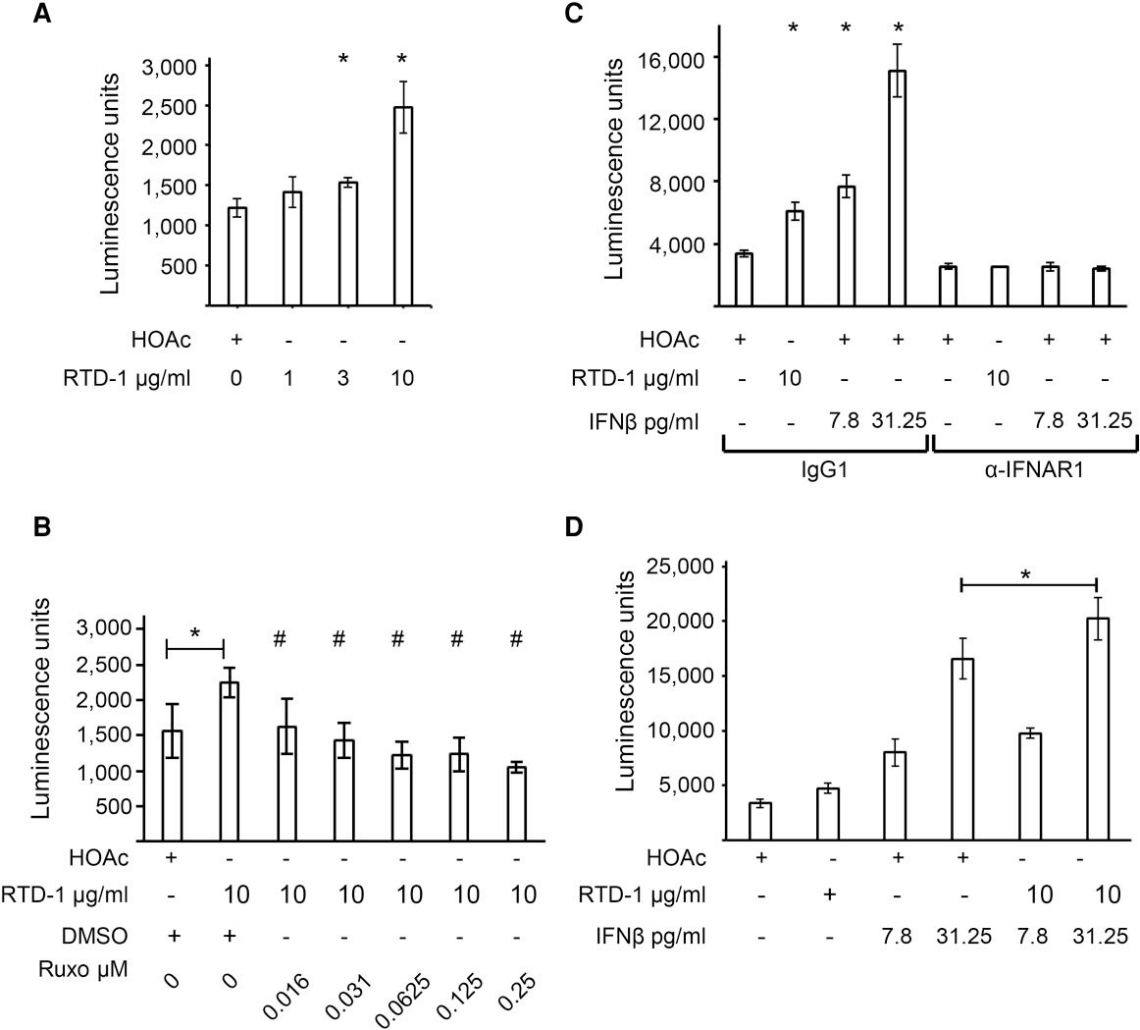
Stimulation of ISRE reporter by RTD-1. The activity of Lucia luciferase secreted in the medium was assayed and the luminescence units are plotted. (A) THP-1 Dual cells were stimulated with HOAc (vehicle) or increasing concentrations of RTD-1. Results are from 3 experiments. *P < 0.05, 2-tailed *t* test, comparison with HOAc-treated sample. Error bars indicate standard deviation. (B) THP-1 Dual cells were pretreated with DMSO or Ruxo as indicated. Assay done in triplicate. *P < 0.05 when compared with HOAc-treated control, 2-tailed *t* test. #*P* < 0.05 when compared with RTD-1 treatment. Error bars indicate standard deviation. (C) THP-1 Dual cells were transferred to RPMI + 1% HI FBS + P/S + normocin medium and either 2.5 μg human IgG1 or anti-IFNAR1 antibodies were added to the culture. Cells were incubated for 2 h, and RTD-1 or IFN-β was added as indicated and the incubation continued for 18 h. Assay was done in triplicate. *P < 0.05 when compared with HOAc-treated control, 2-tailed *t* test. Error bars indicate standard deviation. (D) THP-1 Dual cells were treated with RTD-1, IFN-β, or both as shown. Assay was done in triplicate. *P < 0.05, 2-tailed *t* test. Error bars indicate standard deviation.

To more directly evaluate the effect of RTD-1 on IFN pathways, the peptide was incubated with THP-1 Dual cells in which activation of the ISRE promoter drives expression of secreted Lucia luciferase. Treatment of THP-1 Dual cells with RTD-1 showed RTD-1–concentration dependent increase in the luciferase reporter activity demonstrating stimulation of the ISRE reporter (Fig. 5A), and this stimulation of ISRE reporter was inhibited in the presence of Ruxo (Fig. 5B). To evaluate the requirement for type I IFN receptor, THP-1 Dual cells were stimulated either with RTD-1 or the positive control IFN-β in the presence of anti-IFNAR1 antibodies or isotype human IgG control antibodies. The stimulation of ISRE activity by RTD-1 and IFN-β was inhibited by anti-IFNAR1 antibodies but not by control IgG, demonstrating a role for type I IFN receptor in RTD-1–mediated stimulation of the ISRE reporter (Fig. 5C). The IFN-β–stimulated ISRE reporter activity was further increased upon costimulation with 10 μg/mL RTD-1 (Fig. 5D), suggesting a common pathway in regulation of ISRE promoter by RTD-1.

**Fig. 6.**
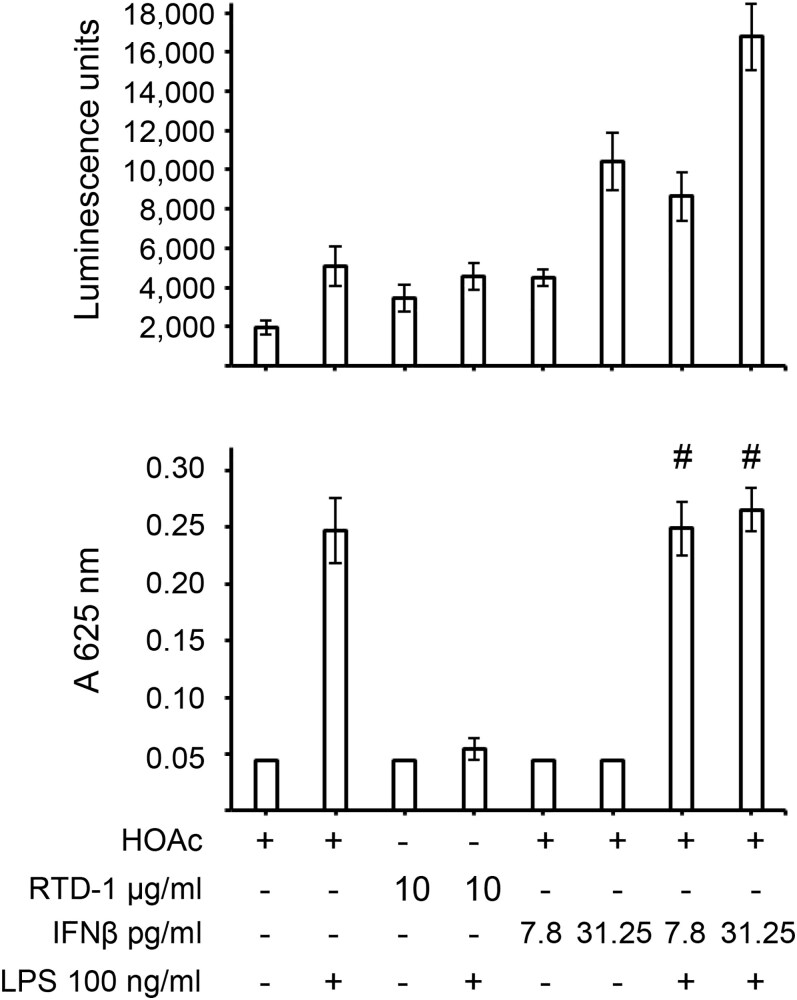
ISRE stimulation activity is distinct from RTD-1–mediated inhibition of NF-κB activation. THP-1 Dual cells were treated with LPS, RTD-1, or IFN-β or vehicle as shown and the medium was assayed for ISRE reporter activity (top) and NF-κB reporter activity (bottom) using Quanti-Luc and Quanti-Blue assays, respectively. Assay done in triplicate. #*P* > 0.05 (nonsignificant) when compared with respect to LPS treated samples, 2-tailed *t* test. Error bars indicate standard deviation.

To test if RTD-1 ISRE stimulation activity is in any way related to its ability to regulate NF-κB activation in immune stimulated cells, we stimulated THP-1 Dual cells with LPS in the presence or absence of RTD-1 or IFN-β and evaluated the stimulation of ISRE and NF-κB activation. As expected, LPS, RTD-1, or IFN-β increased ISRE activity in THP-1 Dual cells on their own. In the presence of LPS no increase in ISRE activity was observed by addition of 10 μg/mL RTD-1; however, an increase in ISRE reporter activity was observed in the presence of LPS and IFN-β (Fig. 6, top). The NF-κB reporter was stimulated by LPS but not by either RTD-1 or IFN-β alone. RTD-1 inhibited LPS-stimulated NF-κB activation as reported previously for differentiated THP-1 cells. In contrast to the ISRE reporter, LPS-stimulated NF-κB activation (Fig. 6, bottom) was unaffected by IFN-β. These results demonstrate that the anti-inflammatory effect of RTD-1 in regulating activation of NF-κB pathway in immune-stimulated cells is distinct from its activation of the ISRE reporter.

### 3.4 RTD-1 inhibits infection of cells by VSV-G and SARS-CoV2-S pseudotyped viruses

**Fig. 7.**
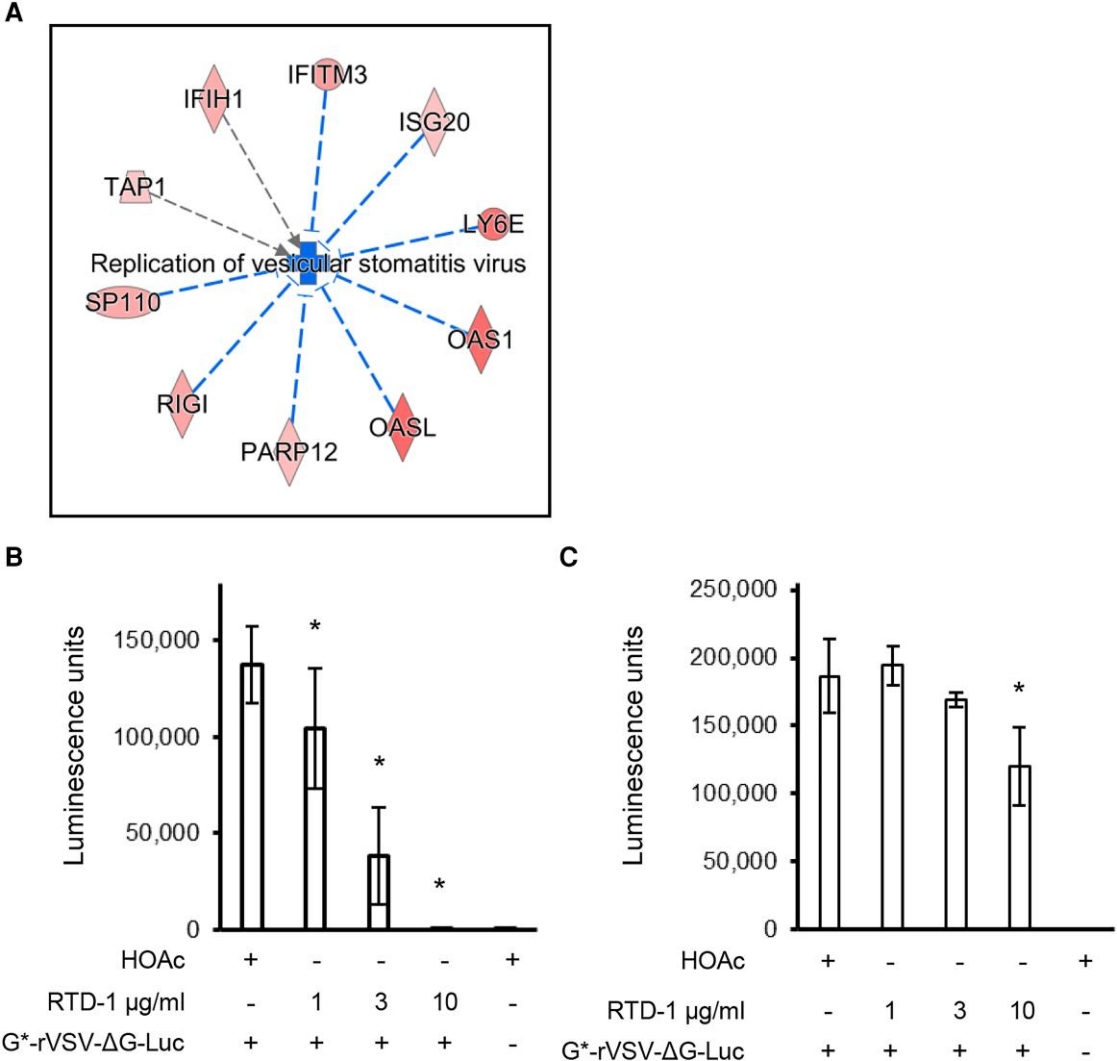
Antiviral activity of RTD-1. (A) Diseases and functions analysis using IPA shows regulation of genes that predict inhibition of replication of VSV. The red color of the genes indicates increased gene expression, blue color indicates inhibition, and the intensity of color corresponds to gene expression. (B) THP-1 cells were incubated with RTD-1 as indicated and G*-rVSV-ΔG-Luc and infection was assayed by luciferase activity. (C) G*-rVSV-ΔG-Luc was preincubated with RTD-1 as shown, diluted 10-fold and used to infect THP-1 cells; infection was assayed using luciferase assay. Results are from 3 experiments. *P < 0.05, 2-tailed *t* test. Error bars indicate standard deviation.

The effects of RTD-1 on monocyte and THP-1 transcriptomes identified several genes implicated in inhibition of VSV (Figs. 3B and 7A). To assess the effects of RTD-1 on VSV infection, we preincubated THP-1 cells with the peptide and then infected with luciferase reporter expressing recombinant VSV pseudotyped with VSV-G (G*-rVSV-ΔG-Luc), with the resulting luciferase activity used as the measure of viral infection. THP-1 cells infected with G*-rVSV-ΔG-Luc for 18 h produced robust luciferase activity demonstrating efficient infection of the cells. Incubation of THP-1 cells with RTD-1 4 h prior to addition of VSV resulted in inhibition of infection in a concentration-dependent manner (Fig. 7B). To test if this reduction in luciferase activity by RTD-1 was due to inactivation of the virus or inhibition of infection, the pseudotyped virus was preincubated for 1.5 h with 1, 3 or 10 µg/mL RTD-1. As shown in Fig. 7C pretreatment of G*-rVSV-ΔG-Luc had no impact on infectivity except at the highest peptide level tested, indicating that direct inactivation of the virus is not the primary mechanism of infection inhibition.

**Fig. 8.**
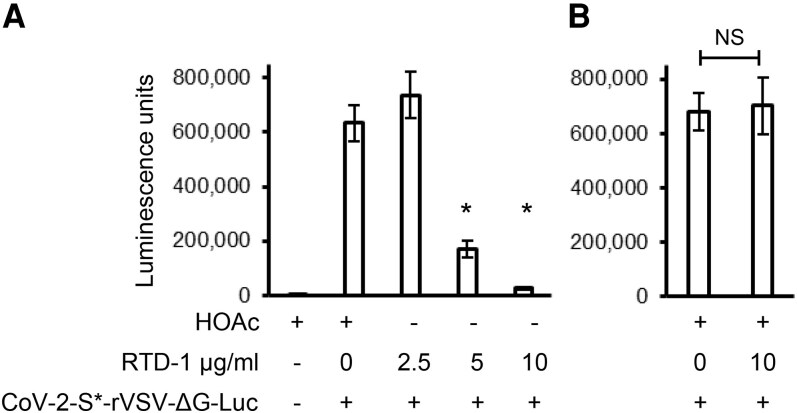
RTD-1 inhibition of SARS CoV-2 S pseudotyped virus infection. (A) Vero E6 cells were incubated with RTD-1 and CoV-2-S*-rVSV-ΔG-Luc as indicated and infection was assayed by luciferase activity. (B) Vero E6 cells were infected with CoV-2-S*-rVSV-ΔG-Luc and 10 µg/mL RTD-1 was added at the end of the incubation and the luciferase activity was assayed. Experiment done in triplicate *P < 0.05, 2-tailed *t* test. Error bars indicate standard deviation. NS, not significant.

To test if RTD-1 inhibition of infection is mediated through the VSV-G glycoprotein in the pseudotyped virus, we evaluated the effect of RTD-1 on infection by SARS-CoV-2 S protein pseudotyped virus. RTD-1 inhibited infection of Vero E6 cells with CoV-2-S*-rVSV-ΔG-Luc pseudotyped virus in a concentration-dependent manner (Fig. 8A). Addition of RTD-1 at the end of infection had no effect on luciferase activity (Fig. 8B), demonstrating that the infection reporter enzyme is not inhibited by the peptide. These results demonstrate that RTD-1 suppresses pseudotyped VSV infection in a manner independent of the envelope glycoprotein.

### 3.5 RTD-1 inhibits SARS-CoV-2 infection

**Fig. 9.**
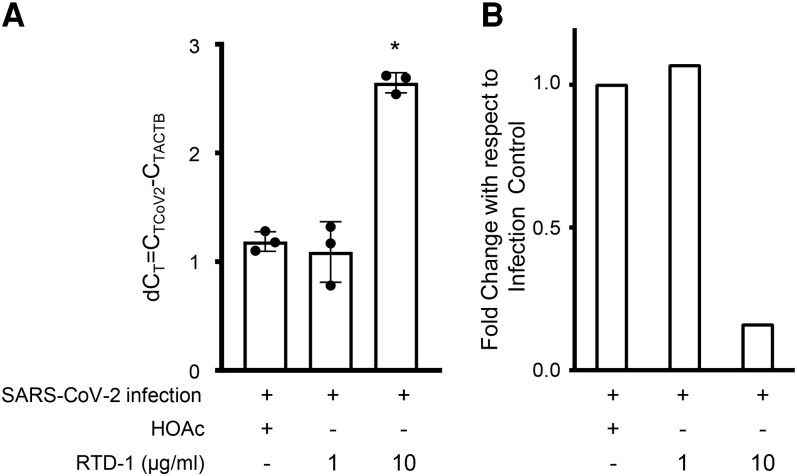
Inhibition of SARS-CoV-2 infection of Calu3 2B4 cells by RTD-1. The effect of RTD-1 on infection of Calu-3 2B4 cells with SARS-CoV-2 was assessed by qRT-PCR using primers for SARS-CoV-2 N gene and normalized to ACTB expression. (A) d_CT_ values, (B) data from panel A expressed as fold change with respect to infection control. Assay was done in triplicate. *P < 0.05, 2-tailed *t* test. Error bars indicate standard deviation.

To further assess the antiviral properties of RTD-1, we tested the effect of the peptide on SARS-CoV-2 infection of Calu-3 2B4 human airway epithelial cells, a line that has been extensively used for SARS-CoV-2 infection studies (THP-1 cells are nonpermissive to SARS-CoV-2 infection). Analysis of SARS-CoV-2 infected Calu-3 2B4 cells by qRT-PCR demonstrated expression of SARS-CoV-2 N gene comparable to host cell ACTB gene expression (Fig. 9A). No SARS-CoV-2 gene product was detected in uninfected Calu-3 2B4 cells (C_T_ value >35). Infection in the presence of 1 µg/mL RTD-1 did not significantly affect SARS-CoV-2 N gene expression. However, at 10 µg/mL RTD-1, there was a statistically significant increase in dC_T_ value and a corresponding decrease in N gene expression relative to untreated infected controls (Fig. 9A, B) demonstrating RTD-1 mediated reduction of infection of Calu-3 2B4 cells.

## 4. Discussion

Here, we used RNA-seq analysis to reveal that RTD-1 stimulates antiviral and IFN pathway gene expression in naïve human monocytes and monocytic THP-1 cells. While IFNs were not differentially expressed at our experimental time point, analysis of the 143 DEGs in 10 μg/mL RTD-1–treated THP-1 cells using IPA showed activation of type I, II, and III IFNs, and IFNL1, IFNA2, IFNG, IFNB1, and IFN-α, were among the top 10 activated upstream regulators (Table S6). The gene expression patterns induced by RTD-1 treatment clearly indicated activation of antiviral and IFN response pathways. The direction of regulation (either activated or inhibited) of upstream regulators (Fig. 3C;  Table S7) and Diseases and Functions categories (Table S8) observed for RTD-1–treated human monocytes and THP-1 cells was similar. Therefore, RTD-1 induced comparable gene expression and signaling pathways in primary human monocytes and THP-1 cells.

RTD-1 stimulated phosphorylation of STAT1 at the Y701 residue in a JAK-dependent manner at an early, 30-min time point (Fig. 4). This suggests a direct cellular action of RTD-1 instead of an indirect mechanism through another pathway. In agreement with the stimulation of antiviral and IFN pathways, RTD-1 stimulated the ISRE promoter in a dose-dependent manner (Fig. 5A). This activation was inhibited by Ruxo (Fig. 5B) and specifically by anti-IFNAR1 antibodies and not by control human IgG1, suggesting a role for type I IFN receptor in the RTD-1–mediated stimulation of ISRE reporter (Fig. 5C). Cotreatment of THP-1 Dual cells with RTD-1 and IFN-β additively increased ISRE stimulation (Fig. 5D), suggesting a common stimulation pathway consistent with the inhibition of RTD-1–mediated ISRE stimulation by anti-IFNAR1 antibodies. This stimulation of ISRE reporter by RTD-1 is distinct from its previously reported inhibition of NF-κB activation in LPS-stimulated cells,^11^ demonstrating a novel role for RTD-1 in regulating diverse cellular signaling pathways (Fig. 6).

Analysis of gene expression pathways indicated activation by RTD-1 of cellular antiviral pathways (Figs. 1C and 3B). Diseases and Functions analysis of gene expression of THP-1 cells treated with 10 μg/mL RTD-1 using IPA identified 8 genes that inhibit VSV replication, a single stranded negative sense RNA virus (Fig. 7A). Of these, DDX58/RIG1,^26^ IFITM3,^27^ ISG20,^28^ Ly6E,^29^ OAS1,^30^ OASL,^31^ and PARP12^29^ are directly involved in inhibition of VSV infection. In agreement with this, RTD-1 inhibited recombinant VSV-G pseudotyped virus infection of THP-1 cells. Preincubation of the virions with RTD-1 only slightly reduced their infectivity, suggesting that inhibition is due to host-directed response at a postentry step. RTD-1 also inhibited infection of Vero E6 cells by SARS-CoV-2 S protein pseudotyped rVSV, demonstrating that this inhibition is not specific to envelope protein. While Vero cells are known to be deficient in type I IFN genes^32^ and in IFN responses,^33^ antiviral responses have been identified in these cells.^34–36^ In Vero and other cell lines, alternate IFN- and IRF3-independent induction of early antiviral responses by phosphorylation of STAT1 at Y701 residue upon activation of the RIG-I/MAVS pathway has been identified,^25^ and it is likely that RTD-1 may recruit this or other noncanonical cell-specific antiviral pathway to mediate its effects.

The Coronavirus Pathogenesis Pathway was predicted to be inhibited in both monocytes and THP-1 cells treated with 10 µg/mL RTD-1. RTD-1 treatment upregulated expression of oligoadenylate synthetase family genes, OAS1, OAS2, OAS3, and OASL, which are activated by double-stranded RNA and stimulate the antiviral activity of RNase L^37,38^ and are involved in response to multiple viruses including SARS-CoV-2.^39–41^ Consistent with this, RTD-1 inhibited the infection of Calu-3 2B4 cells with SARS-CoV-2 (Fig. 9). Retrocyclins, which are human θ-defensin pseudogene-derived cyclic peptides, were also reported to destabilize SARS-CoV-2 S protein^42^ and inhibit SARS-CoV-2 infection^43^ in cellular assays. However, in those studies the host responses to retrocyclin treatment were not investigated.

While this study identified antiviral genes and pathways regulated by RTD-1, including STAT-1 phosphorylation and stimulation of the ISRE promotor, we have yet to identify the RTD-1 receptor(s) or an integrated mechanism that mediates regulation of gene expression and inhibition of VSV and SARS-CoV-2 infection. In a previous study, RTD-1 reduced mortality in an in vivo mouse model for SARS-CoV-1 infection by alleviating pulmonary inflammation and regulating lung cytokine levels,^10^ consistent with the regulation of host gene expression pathways by RTD-1 reported here. Other studies have demonstrated that RTD-1 inhibits HIV-1 by inactivation of the virus and/or inhibiting viral entry and replication.^44–46^ Rhesus theta defensins inhibited entry of HSV-2 virus by binding *O*- and *N*-linked glycans on the glycoprotein B2 receptor.^47^ RTD-1 inhibits human papillomavirus infection by binding viral capsid and inactivating the virus.^48^ Interestingly, IFN-γ–mediated gene expression induced by BCG intravenous injection or by intranasal recombinant IFN-γ application is protective in a mouse model for SARS-CoV-2 infection,^49,50^ consistent with identification of IFNG as an activated upstream regulator in RTD-1–treated cells.

The finding that RTD-1 induces antiviral and IFN pathways in naïve and infected cells reveals a new immunoregulatory property of θ-defensins. The current findings should facilitate the further characterization of θ-defensin–induced antiviral and immunoregulatory responses in in vitro and in vivo models of viral infection.

## Supplementary Material

qiaf150_Supplementary_Data

## Data Availability

Data for THP-1 cells and monocytes has been submitted to Gene Expression Omnibus database with accession numbers GSE278567 and GSE278568, respectively.
